# CO-CREATING A VIRTUAL REALITY PROGRAM WITH PEOPLE LIVING WITH DEMENTIA IN LONG-TERM CARE

**DOI:** 10.1093/geroni/igae098.1079

**Published:** 2024-12-31

**Authors:** Lillian Hung, Ben Mortenson, Angelica Lim, Jennifer Boger, Joey Wong, Christine Wallsworth, Jim Mann, Lily Wong

**Affiliations:** University of British Columbia, Vancouver, British Columbia, Canada; University of British Columbia, Vancouver, British Columbia, Canada; Simon Fraser University, Burnaby, British Columbia, Canada; University of Waterloo, Waterloo, Ontario, Canada; University of British Columbia, Vancouver, British Columbia, Canada; University of British Columbia, Vancouver, British Columbia, Canada; University of British Columbia, Vancouver, British Columbia, Canada; University of British Columbia, Vancouver, British Columbia, Canada

## Abstract

People living with dementia in long-term care (LTC) are a diverse group with varied physical and cognitive abilities and backgrounds. Previous research has shown that virtual reality experiences can bring joy and foster the health and well-being of people living with dementia. A tailored approach based on cultural preferences, needs, abilities, disabilities, and contextual limitations is needed to foster practical implementation, uptake, and sustainability. This qualitative study harnessed the experiential knowledge of people with dementia, care partners and frontline staff to co-build a novel Virtual Reality Program in two Canadian LTC homes. We conducted focus groups and interviews with 10 residents living with dementia, 10 family care partners and 12 staff to explore their preferences on the video content and delivery methods. We also explored participants’ experiences in the co-creation process. Our interdisciplinary team, including people with dementia and family partners, researchers, clinicians, and trainees, analyzed the data thematically. We identified four themes: 1) Significance of culturally relevant and diverse videos to address heterogeneous population, 2) Acknowledgement of residents’ autonomy and choice in the co-creation process, 3) Feelings of satisfaction and motivation through contributing and learning, and 4) Appreciation of a respectful co-design environment. The results offer useful insights to inform future directions in co-designing safe and accessible virtual programs with people living with dementia in LTC research.

